# Leveraging selection for function in tumor evolution: System-level cancer therapies

**DOI:** 10.1093/emph/eoaf022

**Published:** 2025-08-18

**Authors:** Frédéric Thomas, Jean-Pascal Capp, Antoine M Dujon, Andriy Marusyk, Klara Asselin, Mario Campone, Pascal Pujol, Catherine Alix-Panabières, Benjamin Roche, Beata Ujvari, Robert Gatenby, Aurora M Nedelcu

**Affiliations:** CREEC/CANECEV, MIVEGEC (CREES) Department, University of Montpellier, CNRS, IRD, Montpellier, France; Toulouse Biotechnology Institute, INSA, CNRS, INRAE, Toulouse, France; CREEC/CANECEV, MIVEGEC (CREES) Department, University of Montpellier, CNRS, IRD, Montpellier, France; School of Life and Environmental Sciences, Deakin University, Waurn Ponds, Victoria, Australia; Department of Cancer Physiology, H Lee Moffitt Cancer Center and Research Institute, Tampa, FL, USA; CREEC/CANECEV, MIVEGEC (CREES) Department, University of Montpellier, CNRS, IRD, Montpellier, France; Institut de Cancérologie de l'Ouest-Saint Herblain, Centre de Recherche en Cancérologie et Immunologie Intégrée Nantes-Angers INSERM UMR1307/CNRS UMR 6075/Université Nantes/Université Angers; CREEC/CANECEV, MIVEGEC (CREES) Department, University of Montpellier, CNRS, IRD, Montpellier, France; Oncogenetic Department, University Medical Centre of Montpellier, Montpellier, France; CREEC/CANECEV, MIVEGEC (CREES) Department, University of Montpellier, CNRS, IRD, Montpellier, France; Laboratory of Rare Human Circulating Cells and Liquid Biopsy (LCCRH), University Medical Centre of Montpellier, Montpellier, France; European Liquid Biopsy Society (ELBS), Hamburg, Germany; CREEC/CANECEV, MIVEGEC (CREES) Department, University of Montpellier, CNRS, IRD, Montpellier, France; School of Life and Environmental Sciences, Deakin University, Waurn Ponds, Victoria, Australia; Department of Cancer Physiology, H Lee Moffitt Cancer Center and Research Institute, Tampa, FL, USA; Department of Biology, University of New Brunswick, Fredericton, NB, Canada

**Keywords:** selection, tumors, evolution, therapy

## Abstract

Current cancer therapies often fail due to tumor heterogeneity and rapid resistance evolution. A new evolutionary framework, ‘selection for function,’ proposes that tumor progression is driven by group phenotypic composition (GPC) and its interaction with the microenvironment, not by individual cell traits. This perspective opens new therapeutic avenues: targeting the tumor’s functional networks rather than individual cells. Real-time tracking of GPC changes could inform adaptive treatments, delaying progression and resistance. By integrating evolutionary and ecological principles with conventional therapies, this strategy aims to transform cancer from a fatal to a manageable chronic disease. Crucially, it does not necessarily require new drugs but offers a way to repurpose existing therapies to impair a tumor’s evolutionary potential. By steering tumor evolution toward less aggressive states, this approach could improve prognosis and long-term patient survival compared to current methods. We argue that leveraging GPC dynamics represents a critical, yet underexplored, opportunity in oncology.

## Teaser

**Significance Statement** Traditional cancer therapies focus on eliminating tumor cells, yet resistance frequently emerges due to tumor heterogeneity and adaptability. We introduce a novel framework based on the recently proposed concept of *selection for function*, emphasizing that tumor progression is shaped by its group phenotypic composition (GPC) and system-level interactions rather than solely by individual cells and mutations. This paradigm shift suggests new therapeutic avenues: instead of targeting individual cells, disrupting oncogenic functional networks that underlie oncogenic GPCs can destabilize tumors and guide their evolution toward less aggressive states. Our approach integrates evolutionary and ecological principles into cancer treatment, offering a strategy to enhance long-term therapeutic success while leveraging existing drugs in innovative ways.

## INTRODUCTION

Therapeutic approaches against cancer have primarily aimed to eliminate tumor cells, either by targeting them specifically, as in targeted therapies and immunotherapies, or by using less selective approaches like chemotherapy and radiotherapy, which have a higher impact on rapidly dividing cells but affect healthy cells to some extent. These methods focus on obliterating cancer cells or disrupting their ability to proliferate or survive; that is, they are directed against the fitness of individual cells. However, these strategies often face limitations, notably due to the cellular heterogeneity within tumors and the rapid evolution of resistant cells driven by the complex interplay between genetic, epigenetic, and environmental factors. While tumor cell populations may sometimes exhibit group dynamics that promote resilience, certain aspects of population dynamics, such as Allee effects (**see glossary**), can also influence tumor persistence by reducing the likelihood of extinction at very low cell densities. Specifically, while Allee effects generally reduce growth in small populations, they may paradoxically support certain cooperative behaviors among surviving cells that could contribute to tumor regrowth post-treatment [1, 2].

**Figure 1:**
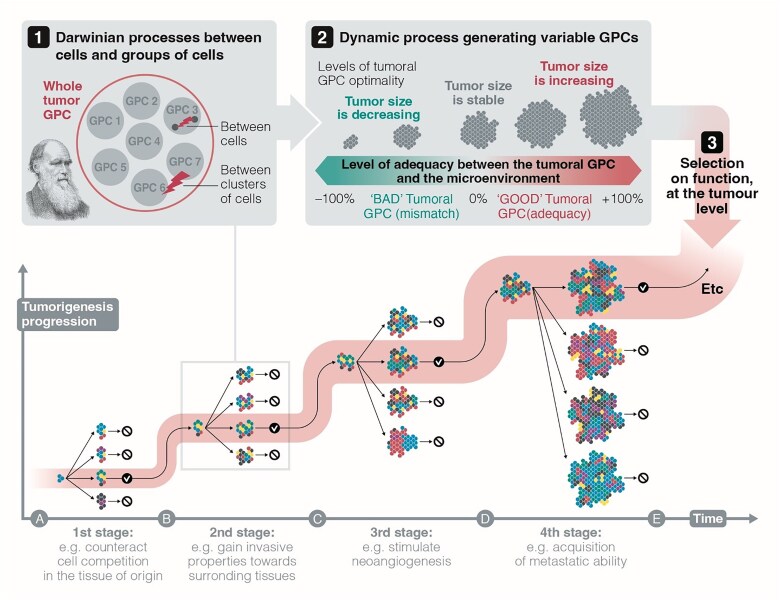
Nested selective processes drive the long-term evolutionary success of a tumor. The example illustrated here depicts the progression toward a malignant tumor (keeping in mind that this is not the only potential pathway as explained in the text). (a) The initial phases of tumorigenesis are influenced by Darwinian processes acting on individual cells and cell groups. (b) These processes continuously create diverse GPCs within the tumor. (c) The emergence of these variable GPCs leads to a second dynamic focused on selection for function, where tumor growth is promoted when the tumor’s emergent properties align with the current microenvironment, influenced by the GPC. Understanding these evolutionary dynamics and the underlying mechanisms is crucial for a comprehensive grasp of tumorigenesis. Adapted from [3].

Recently, a new evolutionary principle governing tumor progression, based on the concept of ‘**selection for function**’ (**see glossary**), has been proposed by Thomas et al. [3] (Box 1  **and**  Fig. 1). This framework suggests that tumor persistence and progression is not solely governed by individual cell-level selection processes acting on random genetic and epigenetic alterations, but rather by the tumor’s ability to form and adjust its **Group Phenotypic Compositions** (GPCs, **see glossary**) in response to selection for function (where ‘function’ refers to any configuration that improves persistence and progression) acting at the tumor level. While previous studies have highlighted the role of the tumor microenvironment (TME) in modulating tumor progression, these models have primarily focused on the selection of individual clones in response to microenvironmental pressures or interactions with other clones or non-tumor cells. In contrast, our perspective shifts the focus from a view in which the TME exerts selective pressures (directly or indirectly) on individual clones, to one where tumors themselves are selected based on their ability to organize functionally advantageous GPCs. This distinction highlights that tumor evolution is driven by selection processes that operate at the system level, rather than being a sum of independent clonal adaptations. Said differently, although the tumor is not a Darwinian individual in the sense of having heritable variation in fitness, we argue that selection can still act at the tumor level to stabilize functional configurations, what we termed ‘selection for function’ (based on Wong et al. [4]), without requiring tumor reproduction and heritability (Thomas et al. [3]). This corresponds to a form of system-level selection that acts on dynamic tumor-level traits, not on cell lineages.

To further clarify how our framework relates to other existing conceptual approaches in cancer research, we provide in Table 1 a comparative synthesis of their respective focus, supporting evidence, potential limitations, and therapeutic implications. While previous studies have extensively documented the critical role of the TME, intercellular cooperation, and niche construction in cancer evolution (including system-level perspectives as articulated by Hanahan and Weinberg [5] and co-evolutionary models such as those proposed by Tabassum and Polyak [6]), our approach builds upon, rather than challenges, this important body of work. In particular, we recognize that these frameworks already describe multiple emergent properties of tumors, including signaling circuitries, functional heterogeneity, and cooperative traits among different lineages. The originality of the present framework lies in its explicit evolutionary formulation of the new concept of ‘selection for function’ acting at the tumor level (as a non-replicative entity), and in its operationalization of the ecological concept of GPC [7] as an integrative analytical tool. Rather than replacing existing concepts, the GPC framework aims to provide an evolutionary and systems-level logic that unifies these multiscale interactions as emergent, dynamic configurations. Furthermore, rather than a rebranding of earlier concepts, GPC serves to unify the spatial, functional, and evolutionary dynamics of tumor systems under a single framework, explicitly centered on the selection for system-level functionality. This perspective allows us to articulate new therapeutic avenues focused on disrupting not only specific cells or molecular pathways, but also the functional organization of tumors themselves as adaptive evolutionary systems.

Box 1. What is ‘selection for function’ in the context of tumor progression?

The classical view of tumor progression focuses on the selection of individual cells that possess survival and proliferation advantages [12, 13]. The ‘selection for function’ view, on the other hand, considers the tumor as a dynamic, complex system where specific cells and groups of cells contributing to the group phenotypic composition (GPC) of a tumor are retained or stabilized based on their functional contribution to the overall survival and growth of the tumor [3]. This view is not just about which cells survive and proliferate more, but also about how they interact, and directly or indirectly affect each other’s fitness within the tumor to promote its overall success. For example, a tumor might grow and be successful (i.e. maintained within the system over time) not because cells divide rapidly, but because it exhibits a specific group composition (e.g. contains groups of cells that promote angiogenesis, thus providing resources to the tumor as a whole). Although this process does not correspond to classical Darwinian selection acting on the tumor as a whole, it reflects an internal sorting and stabilization of group-level functions that promote tumor persistence, and indirectly increase cell-level fitness. Selection for function proposes a shift in perspective by considering the tumor as a complex system where selection acts on both individual cells and GPCs based on their contribution to the persistence or progression of the tumor. This means that even cells with low individual direct fitness advantages can be selected if they contribute to the tumor’s survival and progression in another way. Mechanistically, the tumor’s ability to organize itself, persist and progress arises from local interactions among diverse cell types, mediated by molecular signals such as cytokines, growth factors, and extracellular matrix remodeling. These interactions can create self-reinforcing feedback loops and spatial compartments that stabilize emergent group-level functions like immune evasion or angiogenesis, without the need for top-down coordination. Beyond its conceptual significance, selection for function at the tumor level has direct therapeutic implications. Unlike conventional strategies that focus on eradicating individual cancer cells based on their mutational profiles, our approach suggests that disrupting functional networks that underlie the tumor GPC can prevent its adaptive resilience to treatment. This perspective offers a novel strategy for improving long-term patient outcomes, which we discuss in detail in later sections.

Importantly, this framework differs from classical group selection. In traditional models of group selection, different groups (e.g. populations, demes, or colonies) vary in fitness and compete over multiple generations, with successful groups reproducing more and transmitting their heritable traits. In contrast, tumors are non-replicating entities: they do not reproduce to form heritable lineages in the Darwinian sense. There is no inter-tumor selection based on heritable group-level traits (**see glossary**). Instead, selection for function refers to selection of configurations (GPCs) that allow tumors to persist and adapt within their current microenvironment. GPCs that confer survival advantages are not transmitted to offspring, but they can, *de novo,* emerge, stabilize, or reconfigure dynamically within tumors depending on environmental conditions and therapeutic pressures. Although tumors are not reproducing entities in the Darwinian sense, the ecological dynamics of tumor emergence and extinction bear similarities to extinction-recolonization processes in metapopulation models. Most nascent proto-tumors go extinct without progressing, akin to failed colonizations. However, under certain conditions, such as the co-occurrence of synergistic mutations or phenotypes that produce a favorable GPC, some tumors persist and progress. This dynamic is reminiscent of Wright’s shifting balance theory, in which local interactions among genetic variants can generate new adaptive peaks that spread through populations. In this light, the persistence of tumors through the stabilization of functional GPCs can be viewed as an evolutionary process driven by system-level selection, akin to the models discussed by Wade and Goodnight [14] and Wade [15], which emphasize the role of extinction and recolonization in group selection. Thus, the tumor does not act as a Darwinian individual and tumors are not selected via differential survival and reproduction within a tumor population, but evolution can still take place as selection favors group-level configurations that confer tumors increased stability and potential to progress in the absence of reproduction and heritability at the tumor level. This conceptualization aligns with generalized models of selection in non-Darwinian systems, as recently proposed by Wong et al. [4]

While our approach could be interpreted as simply disrupting communication and cooperation among cancer cells, we argue that the concept of GPC captures a broader and more integrated perspective. GPCs encompass not only the individual cellular components and their interaction (e.g. communication or cooperation) but also their emergent collective properties, such as angiogenesis, immune evasion, or extracellular matrix remodeling, which result from coordinated functions across heterogeneous subpopulations. Focusing solely on interactions may overlook the importance of specific phenotypic configurations that emerge from the spatial and functional organization of subgroups of cells. In contrast, the GPC concept provides a system-level view of the tumor as an evolving network of functional units, allowing for therapeutic targeting of both subpopulations (the nodes), interactions (the edges), and their emergent system-level behaviors. Unlike conventional eco-evolutionary frameworks that interpret tumor organization as an emergent consequence of clonal dynamics, public good production, and coevolution with the microenvironment, the *selection for function* framework posits that certain *combinations of phenotypes and interactions*, GPCs, are preferentially maintained because they contribute to tumor-level functional coherence (e.g. coordination of angiogenesis and immune evasion). These configurations may persist or re-emerge *de novo* even as the underlying cellular players change, suggesting that selection operates not only on individual traits or clones but on *emergent collective functionality*. This view supports the idea that tumors may stabilize around specific organizational ‘attractors’, favored GPCs, and opens new therapeutic avenues aimed at disrupting their functional architecture.

To improve conceptual clarity, we propose to restrict the use of the term phenotype to traits expressed at the cellular level, encompassing both autonomous features (e.g. proliferation rate, apoptosis resistance) and interactive ones (e.g. cytokine secretion, expression of adhesion molecules). Complex cellular programs such as EMT (epithelial-to-mesenchymal transition) can be viewed as composite phenotypes involving multiple traits. In contrast, higher-level outcomes such as angiogenesis, immune evasion, or metastasis are more appropriately described as emergent processes, arising from the coordination of diverse cell phenotypes within a given GPC. In this framework, selection for function does not act directly on these emergent processes as isolated units, but rather on the underlying multicellular configurations (GPCs) that enable such coordinated behaviors and confer systemic advantages (e.g. enhanced stability, adaptability, or novelty). Selection thus operates both on individual cells bearing traits that contribute to collective outcomes and on group-level configurations that improve tumor-level persistence and progression. Experimental approaches could help disentangle selection pressures acting on individual traits (nodes), interactive traits (edges), or emergent properties of specific GPCs (networks). This perspective extends earlier conceptualizations of the tumor as an *extended phenotype*, by emphasizing the selection of group-level architectures that foster oncogenic function and systemic resilience [16].

One important theoretical question that arises from this framework is whether the stability of oncogenic GPCs could be undermined by the invasion of selfish mutants, a classical challenge in the maintenance of cooperative groups. Tumors, characterized by extreme genetic diversity and rapid mutation rates, would appear particularly prone to this risk. And this aspect could, at least theoretically, explain the failure of some tumors to progress or even their regression. However, several features of tumor ecology could mitigate this vulnerability. First, many of the functions maintained by GPCs, such as angiogenesis, immune evasion, or extracellular matrix remodeling, produce collective benefits that are essential for the short-term survival of all tumor cells, including potential cheaters. The harsh and rapidly changing tumor microenvironment often creates conditions in which cooperative behaviors become indispensable. Second, tumors differ from classical group selection systems in that they exhibit high cellular plasticity and frequent reshuffling of cell populations, which can buffer against the spread of non-cooperative clones. Finally, functional redundancy within GPCs, where multiple clones contribute to essential functions, limits the risk that a few selfish mutants could entirely collapse the system. These characteristics suggest that while GPCs are inherently dynamic and potentially unstable, their vulnerability to cheating can itself be exploited therapeutically, by deliberately disrupting key cooperative functions or amplifying internal conflicts within the tumor.

**Table 1:** Comparison of different conceptual frameworks in cancer research.

Conceptual framework	Main focus	Supporting evidence	Potential limitations	Therapeutic implications
Classical cell-centered view [1, 2]	Intrinsic properties of individual cancer cells (mutations, proliferation, survival)	Extensive experimental data; models of clonal evolution (Nowell 1976); many targeted therapies developed accordingly	Does not explain tumor resilience despite high heterogeneity; neglects collective behaviors	Targeted therapies, chemotherapy, immunotherapy focused on cell elimination
Tumor microenvironment (TME)-centered view [3, 4]	Role of external factors: fibroblasts, immune cells, vasculature, ECM	Very well established; supported by numerous preclinical and clinical studies	Often still focused on cell-level responses to external pressures	Therapies targeting angiogenesis, immune checkpoints, TME remodeling
Intra-tumor cooperation [5, 6]	Cooperation between cancer cells for public goods production (e.g. growth factors)	Experimental evidence of cooperative behaviors; defector cell strategies tested in vitro/in vivo	Cooperation often modeled in simple systems; destabilization by defectors not always feasible	Therapeutic introduction of engineered defectors; disrupting cooperation
Group Phenotypic Composition (GPC)-based view (this work)	System-level organization of phenotypically diverse subpopulations and their interaction networks within tumors	Supported by conceptual models; indirect clinical evidence (e.g. failure of cell-killing-only strategies); observed resilience via functional redundancy	Requires further empirical validation; complex to monitor and model in patients	Therapies targeting functional networks, combination/sequential therapies, system destabilization, GPC misalignment

1. Hanahan D, Weinberg RA. Hallmarks of Cancer: The Next Generation. Cell. 2011;144: 646–674. https://doi.org/10.1016/j.cell.2011.02.013,

2. Greaves M, Maley CC. Clonal evolution in cancer. Nature. 2012. https://doi.org/10.1038/nature10762,

3. Quail DF, Joyce JA. Microenvironmental regulation of tumor progression and metastasis. Nature Medicine. 2013;19(11):1423–37. https://doi.org/10.1038/nm.3394,

4. Junttila MR, De Sauvage FJ. Influence of tumour micro-environment heterogeneity on therapeutic response. Nature. 2013;501(7467):346–54. https://doi.org/10.1038/nature12626,

5. Archetti M, Pienta KJ. Cooperation among cancer cells: applying game theory to cancer. Nature Reviews Cancer. 2019;19(2):110–117. https://doi.org/10.1038/s41568-018-0083-7,

6. Axelrod R, Axelrod DE, Pienta KJ. Evolution of cooperation among tumor cells. Proceedings of the National Academy of Sciences of the United States of America. 2006;103:13474–9. https://doi.org/10.1073/pnas.0606053103

Although this approach encompasses aspects associated with TME, it moves beyond viewing the TME as an external modulator acting on individual clones and instead positions tumors as evolutionary entities whose progression and stability depend on the organization and optimization of functional networks (**see glossary**) rather than on individual cellular traits alone [7]. Our view does not contradict classical views on tumor progression; instead, it integrates an additional layer of complexity, helping to explain why some tumors remain stable (i.e. capable of maintaining their growth, structure, and functional organization over time) despite genomic instability and heterogeneity as well as environmental variability. A key challenge for this conceptual shift is to experimentally distinguish between Darwinian selection acting on individual cancer cells and selection for function acting on GPCs; note that the latter is different from classical group selection in the sense that these groups do not reproduce or express heritable variation in fitness. While tumors, including metastatic ones, can produce ‘offspring’ and colonize distant sites, through cell proliferation and dispersal, they do not constitute Darwinian individuals in the classical sense. That is, they do not form discrete, reproducing lineages with heritable variation in fitness, subject to group-level selection across generations. In contrast to classical group selection (similar to multi-level selection type 2), which relies on differential reproduction and heritable variation among groups, our framework posits that selection for function operates within tumors as dynamic systems, stabilizing emergent configurations (GPCs) that enhance the persistence and adaptability of the tumor. Metastatic progression can thus be understood as a manifestation of selection for function, favoring phenotypic compositions that facilitate dispersal, survival in the bloodstream, immune evasion, and colonization, rather than as evidence for group-level Darwinian reproduction (see [8]). This distinction underscores that selection for function acts on system-level configurations within tumors, not between tumors as competing evolutionary units.

Because many treatment effects impact both individual fitness and group persistence (and the two levels are inter-connected), teasing apart the level on which the treatment acts remains difficult but not intractable. Several experimental strategies can help address this. First, competition experiments using tumor spheroids or organoids composed of mixed clones (e.g. cooperative vs. non-cooperative phenotypes) could be used to test whether tumors with oncogenic GPCs outcompete those composed of individually fitter but non-cooperative cells, particularly under stress conditions such as hypoxia or nutrient limitation. Second, perturbation experiments that selectively disrupt functional interactions (e.g. angiogenesis, ECM remodeling) without directly impairing cell viability could reveal whether tumor regression results from GPC destabilization rather than direct clonal elimination. Moreover, recent studies suggest that group-level dynamics, including non-cell autonomous interactions between subclones, may contribute to the emergence of treatment resistance, beyond the classical model of clonal expansion of pre-resistant lineages [9, 10]. Third, recent advances in spatial transcriptomics (**see glossary**) and single-cell lineage tracing now allow the decoupling of clonal ancestry from contributions to the functioning of the system (see for instance [11]). By tracking whether subclones with low intrinsic fitness persist due to their role in supporting tumor-level persistence, one could provide evidence for selection operating on GPCs. While these strategies remain technically challenging, they offer promising routes to empirically investigate selection for function in tumors.

GPC can change rapidly because cancer cells, unlike normal cells, exhibit a high mutation rate and phenotypic plasticity, allowing them to rapidly acquire new traits through both genetic and epigenetic mechanisms of inheritance. Thus, within tumors, different individual cell-level adaptations can produce phenotypically heterogeneous, dynamic, loosely organized distinct groups that collectively adjust to local environmental selection forces. Consequently, although individual cancer cells remain the reproductive unit of selection, the group dynamics can affect the fitness of each individual cell within the group and contribute to tumor stability or progression (**see**  Box **2** for examples). In other words, rather than viewing the tumor as a mere collection of independent cells, we consider it as a complex, cooperative emergent system where within group-level interactions enhance the survival and dynamic persistence of the tumor. This focus on the collective behavior of cells shifts the therapeutic target from individual cells to the group dynamics that underpin tumor resilience.

Capp et al. [7] suggested that complex tumors that exhibit specific oncogenic GPCs (i.e. GPCs able to sustain tumor growth and to reorganize in response to microenvironmental factors as well as therapies) are the ones that survive (i.e. do not go extinct) and progress. In other words, they are ‘selected’ (relative to other tumors without the appropriate GPC or the ability to adjust their GPC) by the virtue of possessing a ‘functional’ GPC. That is, they are ‘selected for function’, where ‘function’ is defined as an overall emerging dynamic property of the tumor system that allows its stability or progression under changing environments and selective pressures (**see** [3–7] and Box 1).

Box 2. Examples of group dynamics enhancing both the fitness of individual tumor cells and the persistence and progression of tumors.

One example of group dynamics at play during tumor progression is the process of angiogenesis. In a nutrient-limited environment, a single cancer cell producing large quantities of angiogenic factors would incur a high metabolic cost, potentially reducing its fitness by diverting resources from other vital functions. However, within a tumor, the production of these angiogenic factors can be distributed among many cells with each member contributing a smaller amount (shared resources/public goods). This reduces the metabolic burden on any single cell while still achieving sufficient angiogenesis to support the entire tumor’s growth. This cooperative production benefits even clones that do not produce the factors (but can contribute in different ways, see below), increasing the collective success of the tumor. Group dynamics are also evident in the production of proteases. Individual cells within a tumor can produce enzymes that degrade the extracellular matrix, facilitating the metastasis of other cells from the tumor (including those that cannot produce the enzyme) and promoting metastasis. Similarly, individual cells can release factors (e.g. TGF-β1, EGF) that can induce the migration and invasion of cells that do not produce such factors but can respond to them, and this interaction can increase the invasion of both clones (e.g. [17]). Such inter-clonal synergistic cooperative interactions can enhance the overall invasive potential of the tumor. That is, the tumor is ‘selected’ for this function (i.e. tumor persists) because of its specific GPC and the complex intra-tumor functional networks. Targeting one or more of the interacting components of this functional network affects more than that subpopulation, but rather the entire system. Another example is the collective production of local lactic acid, which alters the tumor microenvironment, making it less hospitable to infiltrating host immune cells. This acidification provides a shared defense mechanism that benefits the entire tumor, including those cells that do not directly contribute to acid production. These examples illustrate how group dynamics and functional networks within the tumor allow for division of labor, optimization of resource use, and shared benefits, ultimately enhancing the fitness and survivability of individual cancer cells as well as the stability and progression of entire tumors. Note that GPCs and resulting group dynamics change throughout tumor progression. Understanding these functional interactions and cooperative strategies is crucial for designing therapies that disrupt these collective processes, potentially weakening the tumor’s overall adaptive capacity. Interestingly, the group-level outcomes observed in tumors, such as cooperative secretion of public goods, immune evasion, and extracellular matrix remodeling, share notable similarities with microbial biofilms. In both systems, phenotypic heterogeneity, spatial organization, and intercellular communication contribute to the emergence of collective functions that enhance persistence under environmental stress. Like biofilms, tumors can exhibit resilience to external attacks (e.g. antibiotics or therapies), structural compartmentalization, and dynamic adaptation. This analogy may offer useful insights into how cooperation evolves and is maintained in heterogeneous systems, and may inspire therapeutic strategies aimed at disrupting system-level cohesion.

**Figure 2:**
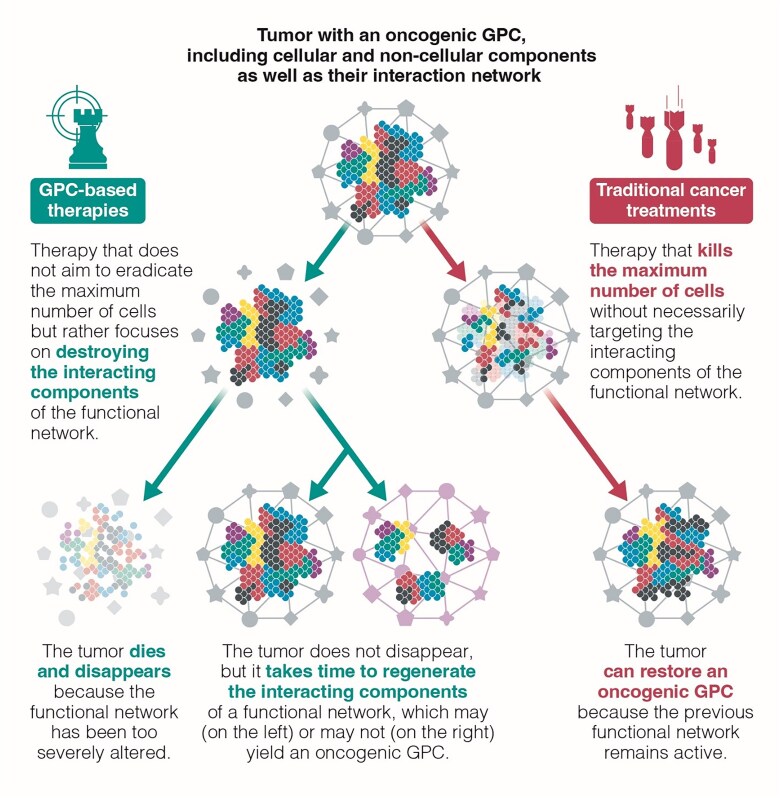
Comparison between cancer treatment approaches: cell destruction vs. functional disruption. This figure illustrates two distinct cancer treatment strategies. The traditional approach (red) focuses on eliminating cancer cells through cytotoxic treatments, such as chemotherapy and radiotherapy. In contrast, our proposed approach aims to target the functional network of the tumor (green), including its microenvironment and signaling pathways, in order to disrupt its stability, progression and ability to evolve.

Understanding the spatial organization and interaction patterns of subpopulations within and among cell groups as well as with the tumor microenvironment can provide insights into the **specific GPCs that can promote tumor progression**. This functional perspective suggests that tumors should not be seen merely as collections of competing individual cells shaped by their microenvironment, but rather as integrated systems where cooperative interactions between subpopulations enable resilience and progression. While many models increasingly recognize the bidirectional dynamics between tumor and TME, early frameworks, particularly those grounded in classical clonal selection, often treated the microenvironment primarily as a selective landscape exerting external constraints on individual cells (e.g. selection pressures due to hypoxia, immune infiltration, or drug exposure) [6, 18]. In contrast, the *selection for function* framework emphasizes that tumor progression is, in part, driven by the maintenance and evolution of oncogenic GPCs. While prior research has extensively characterized how TME influences individual cell adaptation, these studies have largely focused on the selection of individual clones within the tumor. This perspective reframes tumor evolution as the stabilization of functional architectures, emerging from interactions between tumor subpopulations and their microenvironment. This shift in perspective has important therapeutic implications, as it suggests that targeting functional networks rather than isolated cellular traits may offer a more effective strategy for disrupting tumor stability and adaptive resistance. These interactions could allow the tumor to grow (and maintain an oncogenic GPC) even when some cells are destroyed by treatments that affect cells indiscriminately. This explains why therapies targeting individual genetic mutations or disrupting isolated cell-TME interactions often fail, because they do not account for the tumor’s ability to reorganize its functional composition. On the other hand, a therapy specifically targeting such oncogenic GPCs would aim to disrupt these system-level functional networks, and destabilize the tumor, by targeting specific groups (nodes in the network) and/or specific interactions. Rather than targeting the fitness of individual cancer cells, this approach would focus on **altering the functional relationships between groups of cells within the tumor, as well as their interactions with the microenvironment** (**see**  Fig. 2). By extending beyond conventional TME-focused strategies, this model suggests that treatment failures may arise due to a lack of focus on disrupting oncogenic GPCs, which serve as the functional backbone of tumor survival. Our approach does not seek to replace traditional paradigms based on genetic mutations or TME interactions but rather to enhance them by incorporating the concept of selection for function. By considering tumors as evolutionary entities shaped by functional network selection rather than just clonal fitness, our framework provides additional explanatory power for why tumors persist despite aggressive treatments. This perspective complements and extends existing models rather than opposing them. By disrupting these functional networks, therapies could weaken the tumor’s collective survival mechanisms, reducing its capacity to adapt and evolve under therapeutic pressure. This strategy not only promises to counteract resistance but could also improve clinical outcomes by rendering the tumor more susceptible to existing therapies. Recent advances in the in-depth characterization of cell–cell interactions in tumors, from single-cell and spatial transcriptomics [19, 20] and highly multiplexed imaging [21], should provide valuable data for hypothesis generation. These hypotheses, once validated, could lay the groundwork for developing tumor level-based strategies.

By interfering with the tumor’s ability to reorganize its GPC and adjust to a new microenvironment, such therapies could prevent tumor progression and the emergence of new, more resistant subpopulations. Specifically, this approach focuses on destabilizing the tumor by affecting many components of this complex dynamic system, including cellular and non-cellular components as well as their interaction network. This evo-eco (**see glossary**) perspective on GPC-based therapies provides new insights into current therapies and suggests alternative treatment strategies that could potentially be more effective and durable. Below, we discuss and provide suggestions on when GPC-based therapy may be more suitable and promising, as well as how tumors can be destabilized by altering their functional networks and interactions with the microenvironment.

### Contexts for applying GPC-based therapy

A therapeutic strategy aimed at destabilizing a tumor’s GPC would be particularly suitable for tumors whose structural and functional complexities limit the effectiveness of traditional approaches involving resection and/or targeting individual cells through chemotherapy or radiation. For instance, this approach is relevant in cases of advanced but localized cancers, where increased tumor heterogeneity as well as complex interactions between the tumor and its microenvironment can be conducive to the development of oncogenic GPCs and metastasis. Additionally, for cancers that have developed resistance to cytotoxic treatments, this strategy can target the functional networks that support the tumor’s resilience. Finally, in cases where clusters of circulating tumor cells (CTCs, **see glossary**) play a role in metastatic spread, targeting the GPC of these clusters could limit their ability to evade the immune system and colonize other sites. Conversely, for small localized tumors, traditional approaches (e.g. surgery followed by radiotherapy or chemotherapy) may be more appropriate.

### Targeting key functional networks within the tumor GPC

‘Successful’ tumors are those able to progress by inducing angiogenesis (which increases their access to nutrients and oxygen), evading immune surveillance (through mechanisms such as natural killer (NK) cells and antibodies), and remodeling the extracellular matrix to facilitate growth and invasion. These are all emergent properties at the group level that collectively benefit both the entire tumor (i.e. contribute to its progression through selection for function) and the individual tumor cells (**see**  Box **2**). While current therapies targeting these essential properties have been developed, they are typically designed with the objective of directly inhibiting tumor growth or eradicating the tumor. Here, we argue that adapting these therapies to focus on destabilizing the GPC regardless of the direct short-term anti-tumor effects presents a promising direction worth exploring. However, many of these therapies, such as angiogenesis inhibitors or immune checkpoint inhibitors, were not originally developed with the explicit aim of targeting or destabilizing GPCs as system-level configurations. Rather, they were implemented to suppress specific tumor-promoting processes or pathways. Their failure to deliver long-term clinical success in some settings may stem from the tumor’s ability to reorganize its functional composition in response to selective pressure. Our framework suggests that GPC-based strategies should not be seen as new drugs *per se*, but as an intentional therapeutic logic that integrates knowledge of tumor organization and evolution. By leveraging existing therapies in ways that deliberately disrupt functional group-level configurations, particularly when applied sequentially or adaptively, we may enhance treatment durability and overcome resistance mechanisms more effectively than classical approaches. While likely not sufficiently effective on their own, therapies targeting essential emergent properties may enhance treatment efficacy when combined with approaches that specifically target individual cancer cells, such as chemotherapy or targeted therapies. These targeted strategies aim to suppress key cellular players or signals (e.g. those stimulating angiogenesis or inhibiting immune cell action) to limit tumor progression. For example, bevacizumab (Avastin), a monoclonal antibody targeting vascular endothelial growth factor (VEGF), is used to inhibit angiogenesis, depriving the tumor of the nutrients necessary for its growth [22]. In our framework, these strategies are successful because they disrupt functional networks within the tumor rather than solely affecting the targeted cells directly (**see**  Fig. 2). From this perspective, some currently used therapies may have achieved success not only by eliminating specific tumor cells but also, perhaps unwittingly, by disrupting the cohesion or function of key GPCs. For example, anti-angiogenic therapies such as bevacizumab not only deprive tumors of nutrients but may also weaken an emergent functional configuration central to tumor persistence. Conversely, therapeutic failures may sometimes result from neglecting critical group-level interactions, such as intercellular cooperation or support from stromal cells, allowing for the selection or emergence of a new functional configuration (GPC) within the tumor in response to therapeutic pressure. While direct empirical discrimination between effects on GPCs versus individual cells remains challenging, emerging data from spatial transcriptomics and liquid biopsies (**see glossary**) offer potential avenues to track such disruptions more precisely. By reframing past clinical results through the GPC lens, our framework provides a system-level explanatory model that can help identify what mechanisms have (or have not) been disrupted, thus guiding future therapeutic strategies with greater precision. Thus, while the idea of targeting traits beyond proliferation, such as interactions, communication, or angiogenesis, is not new, and some of the cited treatments have existed for over a decade, our contribution lies in proposing a unifying conceptual framework that explicitly organizes these efforts under the umbrella of system-level resilience. What has been lacking, we argue, is not the therapeutic intuition, but a theoretical model that integrates these targets into an evolutionary logic centered on GPC and its emergent properties that promote tumor resilience and progression. In addition, the rarity of true sequential and adaptive therapies may reflect not only practical constraints such as lack of biomarkers or real-time data, but also the absence of a framework that guides treatment design based on the evolution of system-level phenotypes. Our model aims to fill that conceptual gap.

**Disrupting other critical nodes (such as tumor-associated macrophages, cancer-associated fibroblasts) in functional networks** may play a pivotal role in altering oncogenic GPCs. For instance, Pamrevlumab (FG-3019) is a connective tissue growth factor (CTGF) inhibitor that interferes with cancer-associated fibroblasts (CAF) activity to reduce extracellular matrix remodeling [23]. Recent studies have also demonstrated the efficacy of antibody-drug conjugates in depleting CAFs, which play a pivotal role in maintaining the oncogenic microenvironment and facilitating tumor progression. For example, Gallant et al. [24] showed that targeting fibroblast activation protein (FAP) with an antibody-drug conjugate can significantly disrupt the extracellular matrix, reducing tumor growth and invasion through CAF depletion. Similarly, Ostermann et al. [25] demonstrated effective immunoconjugate therapy by targeting a serine protease expressed in tumor fibroblasts, resulting in significant tumor reduction in cancer models. Furthermore, the evolving use of ADCs (Antibody-Drug Conjugate) targeting the ErbB/HER family of receptor tyrosine kinases, as discussed by High et al. [26], illustrates the therapeutic potential of this strategy not only in targeting tumor cells but also in modulating the tumor microenvironment.

**Blocking the molecular signals** that allow cancer cells to communicate (e.g. TGF-β, EGF; **see**  Box **2**) and acclimate to changes in their environment, as well as disrupting their interaction/cooperation with stromal cells, would also destabilize the tumor GPC and/or misalign it with the microenvironment required to develop and maintain an oncogenic state (discussed below). Single-cell RNA sequencing and spatial transcriptomic technologies are now used to infer cell–cell interactions among cancer cells or with stromal cells thanks to the expression of ligand and receptor genes in possible sender and receiver cells [27]. This should extend the range of relevant molecular signals that can be targeted for this purpose. Furthermore, combination therapies affecting multiple nodes and/or signals can be even more effective (**see**  Box **3**). For example, the TGF-β and EGF/EGFR pathways can cross-talk, including through TGF-β inducing the over-expression of EGFR and EGF ligands in other TGF-β responsive (but not producing) cells without constitutively mutated or over-expressed EGFR [28, 29]. Thus, EGFR inhibitors (Erlotinib/Tarceva**,** Osimertinib) could block cancer cell signaling, preventing the proliferation and survival of cells with wild-type EGFR [30], and affect the tumor GPC.

Box 3. Combination therapies to disrupt oncogenic GPCs.

Employing combination strategies that block multiple functions simultaneously can contribute to the destabilization of tumors and negatively affect their oncogenic status or potential. Such combination therapies should aim to block the molecular signals and the components of the signaling pathways that enable cancer cells to cooperate, thus preventing the emergence of new adaptive strategies within the tumor. For example, therapies targeting cell signaling pathways (such as EGFR inhibitors) can be combined with those affecting tumor-stroma interactions, making it harder for cells to cooperate and evolve under treatment pressure. For instance, FG-3019 (Pamrevlumab), a monoclonal antibody targeting connective tissue growth factor (CTGF), has been shown to disrupt the interaction between cancer cells and cancer-associated fibroblasts (CAFs), which are key players in remodeling the extracellular matrix and promoting tumor growth [31, 32]. Additionally, Losartan, an angiotensin II receptor blocker, has been found to modify the tumor microenvironment by reducing the density of CAFs and normalizing the extracellular matrix, enhancing the delivery of chemotherapeutic agents [33, 34]. Moreover, FAP (fibroblast activation protein) inhibitors, such as Sibrotuzumab, target FAP expressed by stromal cells, aiming to disrupt the supportive role of these cells in tumor progression. Therapies targeting angiogenesis, such as Bevacizumab (an anti-VEGF monoclonal antibody), are designed to disrupt the tumor’s ability to recruit blood vessels. Similarly, therapies targeting the immune evasion mechanisms of tumors, such as immune checkpoint inhibitors (e.g. Pembrolizumab targeting PD-1), work by reinvigorating the immune system’s ability to attack the tumor. Additionally, the use of matrix remodeling inhibitors, such as Marimastat, aims to inhibit the activity of matrix metalloproteinases (MMPs) to prevent cancer cell invasion and metastasis. These therapies, by targeting different functional networks within the tumor microenvironment, have shown promising results in destabilizing GPCs. Also, anti-angiogenic agents could be combined with treatments that affect metabolic pathways or immune-modulating therapies to create a multi-faceted attack on tumor functionality. While our theoretical framework introduces a novel perspective, its translation into therapeutic applications does not inherently depend on developing new drugs. Instead, we propose that existing treatments can be repurposed within innovative strategies designed to disrupt the tumor’s evolutionary trajectory, ultimately enhancing prognosis and improving patient survival compared to conventional approaches. Leveraging systems biology approaches to model these interactions could further refine and optimize such therapeutic strategies. Furthermore, we suggest integrating ecology-based strategies into the traditional demographic (cell-based) treatment approaches. Importantly, this also requires focus on strategically combining therapies. However, simultaneous administration of demographic and ecological therapies (the typical approach currently) may be less effective than a sequence of treatments (**see**  Box 4) in which the adaptive mechanisms that allow cancer cell survival to the initial treatment agent(s) renders them more vulnerable to follow-on treatment, dynamics termed ‘evolutionary double bind’ [35, 36] (**see glossary**). Indeed, simultaneous administration of demographic therapies (e.g. cytotoxic treatments targeting individual cancer cells) and ecological therapies (e.g. treatments targeting the tumor microenvironment or stromal interactions) can lead to conflicting selective pressures within the tumor. This may result in the rapid evolution of resistant subpopulations. For example, combining a cytotoxic agent like chemotherapy with an anti-angiogenic therapy (such as Bevacizumab) could initially shrink the tumor, but may also select for cells that can survive under hypoxic conditions, leading to more aggressive, resistant clones. In contrast, a sequential approach, starting with a cytotoxic agent to reduce tumor size and genetic diversity, followed by a second phase targeting the tumor’s adaptive strategies (such as revascularization or immune evasion), allows for more precise targeting of the tumor’s vulnerabilities at different stages of its evolution, thereby reducing the likelihood of resistance. Finally, Gatenby et al. [1] recently proposed a therapeutic strategy inspired by extinction biology. This approach mirrors major extinction events in the natural world, such as the meteorite impact that contributed to the demise of the dinosaurs. After the initial devastation, these catastrophic events can lead to significant reductions in population size and genetic diversity, often fragmenting populations. Subsequently, smaller, otherwise inconsequential factors become critical in driving the species to extinction. In the context of cancer, a similar strategy could be employed: short-term neoadjuvant chemotherapy would serve as a first-line treatment to substantially reduce tumor burden and genetic diversity. Lighter, targeted treatments aimed at altering GPCs would then be used to complete the eradication of the tumor.

While not yet being ready for clinical translation, another GPC-based therapeutic option involves not only targeting oncogenic GPCs but also actively promoting and providing selective advantages to clones that contribute to develop a non-oncogenic GPC (see for instance [37, 38] for examples of non-oncogenic GPCs). This approach aims to create conditions within the tumor and its microenvironment that favor the emergence and persistence of non-oncogenic phenotypes, thereby ‘guiding’ the tumor’s evolutionary trajectories towards a less aggressive and more manageable state. For instance, therapies could be designed to alter the microenvironment in a way that supports metabolic or signaling pathways typical of non-oncogenic cells, effectively discouraging either the proliferation of cells that would otherwise drive tumor progression or the plasticity associated with cell invasion and migration. By doing so, such treatments could selectively promote the growth of cells that are less likely to metastasize or resist therapy. For example, therapies could target the stabilization of the extracellular matrix or manipulate immune modulatory pathways to foster an environment where non-oncogenic cell populations thrive, reducing the chances of aggressive clonal expansion. Moreover, combining this strategy with metabolic reprogramming, such as preventing tumor acidification or promoting oxidative phosphorylation in cancer cells, could further shift the evolutionary dynamics towards less malignant phenotypes [39–41].

This strategy represents a shift from the traditional approach of exclusively targeting oncogenic cells to a more nuanced method of steering the evolutionary dynamics within the tumor. By encouraging the selection of cells that do not contribute to tumor progression, it may be possible to prevent the emergence of aggressive or resistant phenotypes. In turn, this could reduce the likelihood of relapse and the development of therapy resistance, which are major challenges in cancer treatment. Furthermore, this approach could be combined with other therapies that destabilize oncogenic GPCs, thereby exerting a twofold effect: weakening the tumor’s capacity for progression while simultaneously enhancing the competitive advantage of non-oncogenic cells. Ultimately, this dual strategy could lead to more effective and durable patient outcomes, leveraging evolutionary principles to keep the tumor in a controlled, non-lethal state.

The strategies we highlighted above require several multidisciplinary approaches. First, omics technologies such as single-cell transcriptomics and metabolomics hold promise for mapping complex interactions between tumor cells and their microenvironment, potentially helping to identify functional networks that sustain oncogenic GPC stability, though their clinical application remains an area of active research. In cases where tumor cell populations fall below critical density thresholds, Allee effects, typically associated with reduced growth at low population densities, may influence group dynamics within the tumor, potentially hindering growth but also fostering cooperative behaviors that enhance survival. Second, the intratumoral microbiome, which plays a critical role in tumor progression (e.g. [42, 43]), can be explored through metagenomics and metatranscriptomics, offering potential new therapeutic targets. Microbial communities, both intratumoral and systemic, can indeed modulate immune responses, influence metabolic pathways, and affect treatment efficacy. Although not composed of tumor cells *per se*, such microbial agents may contribute to tumor-level emergent properties and can thus be considered peripheral but significant contributors to the GPC. Their presence or absence could enhance or disrupt emergent tumor properties, such as immune suppression or metabolic adaptation, further illustrating the relevance of viewing tumors as functionally integrated systems. Third, artificial intelligence algorithms and predictive modeling can be used to analyze GPC dynamics in real-time, enabling more precise therapeutic adjustments. Finally, integrating multimodal analyses combining genomics, transcriptomics, and imaging provides a comprehensive view of tumor interactions, facilitating the targeting of key nodes to disrupt oncogenic GPCs.

### Functional networks and GPCs in circulating tumor cell clusters

In addition to targeting tumors, similar strategies can be developed against CTC clusters, which are essential players in cancer progression and metastasis as they transport genetic and phenotypic diversity from primary tumors to secondary sites. CTC clusters operate within functional networks that encompass molecular pathways driving proliferation, survival, and resistance to therapy, thereby enabling these cells to endure the harsh environment of the bloodstream [44]. These networks are strongly influenced by the phenotypic composition of CTC clusters, which vary in cellular markers, metabolic activity, and resistance mechanisms. CTC clusters serve as functional units that enhance cell survival and support immune evasion in circulation [45, 46], offering a substantial survival advantage over single CTCs [47, 48]. Studies show that these clusters exhibit higher metastatic potential due to their collective behavior, with enhanced expression of mesenchymal markers like Vimentin and epithelial adhesion molecules such as EpCAM, facilitating strong intercellular adhesion and coordination [49, 50]. Additionally, CTC clusters shift towards glycolytic metabolism, which aids rapid adaptation to hypoxic conditions encountered in the bloodstream [51] and associate with non-cancer cells as platelets, which might aid in their extravasation [52]. Therapeutically, targeting GPCs within CTC clusters offers a promising strategy to interfere with key functional networks, potentially reducing metastatic spread. Key signaling pathways, including TGF-β and HIF-1α, drive the formation, survival and extravasation of these clusters, representing potential therapeutic targets [52, 53]. By using liquid biopsy technology (**see glossary**) to monitor the evolution of CTC GPCs, clinicians could tailor treatments dynamically, aiming to disrupt CTC clusters and hinder metastasis, potentially shifting tumor evolution toward less aggressive phenotypes [54–56].

### Targeting the interaction of GPC with the microenvironment

The GPC can exhibit oncogenic properties that allow tumor progression; however, this is not a fixed state but rather a combination that must be constantly adjusted to the current microenvironment [7]. Said differently, it is important to consider the match between the GPC and the microenvironment to understand why and how a given GPC can be oncogenic and allows a tumor to grow and progress. Therefore, alongside the option of destabilizing the tumor’s GPC (discussed above), modifying the microenvironment can also destabilize tumors by disrupting the GPC/microenvironment coupling responsible for tumor growth. In other words, therapies can be developed to create a mismatch between the tumor GPC and its surrounding microenvironment.

Box 4. Sequential therapies to prevent GPC optimization.

The rapid evolution of tumors under treatment pressure is one of the greatest challenges in the fight against cancer. Each treatment exerts specific Darwinian selection forces on cancer cells, favoring the survival of populations of cells that are or can rapidly adapt to treatment. This phenomenon typically leads to the emergence of therapy-resistant tumors, which significantly limits available treatment options. Sequential therapy aims to address this issue by alternating treatments, targeting different tumor functions at specific intervals, to prevent the tumor from stabilizing into a resistant and aggressive GPC. From a GPC-informed perspective, the goal is not only to kill sensitive cells, but to prevent the tumor from organizing or reorganizing its GPC into an oncogenic state. For instance, the sequential use of cisplatin and metformin, although not originally designed with GPCs in mind, can be reinterpreted through this lens: cisplatin induces DNA damage and cell death, weakening proliferative subclones, while metformin disrupts metabolic cooperation and energy production [66], further destabilizing group-level properties. When used in sequence, these drugs may impair both cellular fitness and collective resilience mechanisms. Other GPC-informed sequential therapies could involve pairing anti-angiogenic therapies with treatments that target glycolytic dependencies or acidosis tolerance. For example, an initial treatment could target cells that promote angiogenesis. By reducing blood flow, this would alter the GPC dynamics, possibly triggering increased glycolytic activity due to hypoxia and enhancing cellular mechanisms to adapt to acidosis. Additionally, the limited blood supply may cause the cells to upregulate receptors for serum growth factors, as they attempt to compensate for the reduced availability of nutrients and signals necessary for survival. Each of these changes creates new potential targets for subsequent treatments. Similarly, a sequence targeting immune evasion followed by one that disrupts stress response pathways (e.g. heat shock proteins, autophagy) could further weaken the tumor’s capacity to preserve an oncogenic GPC.

A critical requirement for such a strategy is the development of real-time adaptive monitoring, which would enable clinicians to adjust the treatment sequence based on the tumor’s GPC evolving response. By integrating data from sequencing and imaging technologies, clinicians could, for instance, detect emerging oncogenic GPCs and modify the treatment plan accordingly.

**Figure 3:**
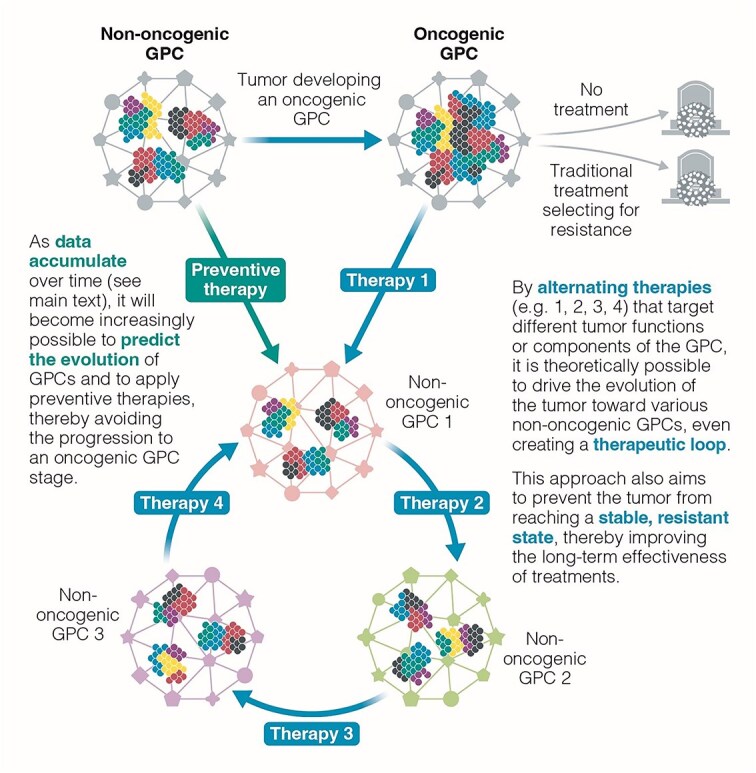
Outcomes of cancer progression under different treatment strategies and practical guidelines for GPC-based approaches. This figure summarizes the potential outcomes of cancer progression under different therapeutic strategies: (a) No treatment (natural progression), (b) classical therapies mainly targeting individual cancer cells (with frequent relapse due to resistance), and (c) innovative strategies leveraging GPC-based approaches aiming to destabilize the tumor system-level networks or to guide the tumor towards less aggressive GPCs. The bottom panel outlines key practical guidelines for defining, monitoring, and applying GPC-based therapies in clinical practice. It emphasizes: • the need for strategic use of existing biomarkers and liquid biopsies to approximate GPC dynamics without exhaustive data collection, • the potential of single-cell and spatial technologies at selected time points rather than continuous monitoring, • the relevance of mathematical and AI-based modeling to predict GPC evolution and guide sequential therapies, • the importance of adaptive clinical trial designs allowing real-time adjustment based on GPC-informed signals, • and the main challenges and limitations to anticipate in applying this framework in real-world settings.

The microenvironment encompasses fibroblasts, immune cells, endothelial cells, as well as microbial cells constituting the tumor microbiome [57, 58] and mycobiome [59, 60], which can all interact with cancer cells and support their growth. The tumor microbiota is especially known to shape spatial and cellular heterogeneity in tumors [61]. It also includes the physical microenvironment, which typically encompasses spatial and temporal variations in blood flow, leading to heterogeneous concentrations of oxygen, serum growth factors, bacterial and cancerous cells metabolites, or extracellular pH. Modifying the microenvironment can significantly affect tumor growth and treatment response. For instance, inhibiting angiogenesis, already part of some therapies, can substantially alter the tumor microenvironment. As mentioned above, treatments that increase the blood buffering capacity (resulting in stabilizing the extracellular pH within tumors) can also produce significant changes in tumor invasion, proliferation, and alterations with immune effectors. In addition, modulators like Losartan, an angiotensin II receptor blocker, can reduce CAF density and normalize the extracellular matrix, facilitating the delivery of chemotherapeutic agents to the tumor [62]. Similarly, targeting stromal components, like cancer-associated fibroblasts which can protect cancer cells from the cytotoxic effects of treatment, may enhance existing treatments [63]. Targeting tumor metabolism is another promising approach. For instance, Lonidamine, which inhibits aerobic glycolysis, could be combined with other treatments aimed at disrupting the tumor’s interaction with the microenvironment, thereby reducing its ability to compensate for energy deprivation [64]. Finally, giving the extensive diversity of mechanisms by which the intratumoral microbiota promote the initiation and progression of tumors by affecting genomic and epigenomic instability, regulating metabolism, promoting inflammation response, avoiding immune destruction, and activating invasion and metastasis [65], its targeting shows great potential in this perspective. Most likely, incorporating such combinations of treatments, either simultaneously or sequentially (**see**  Box **4**  **and**  Fig. 3), will require patient-specific strategies guided by appropriate mathematical models and well-developed biomarkers.

Immunotherapy has emerged as a powerful approach in cancer treatment, not only for its ability to target and eliminate cancer cells but also for its capacity to reshape the tumor microenvironment. An emerging strategy is to combine immune checkpoint inhibitors like Nivolumab (PD-1 inhibitor) with targeted agents disrupting tumor metabolic pathways, aiming to simultaneously impair immune evasion and metabolic dependencies [67]. This combined approach can reduce the adaptability of the tumor by attacking both immune escape and survival strategies. One example is Pembrolizumab (Keytruda), a PD-1/PD-L1 inhibitor, which reinvigorates the immune system’s ability to attack cancer cells by blocking immune evasion mechanisms [68]. By enhancing the immune system’s natural ability to detect and destroy malignant cells, immunotherapies such as immune checkpoint inhibitors and CAR-T cells can significantly alter the dynamics between cancer cells and their surrounding environment. These therapies disrupt crucial interactions between tumor cells and stromal components, such as CAFs and tumor-associated macrophages, which play a key role in supporting tumor growth and immune evasion [69]. By modulating key signaling pathways, such as the PD-1/PD-L1 axis or CTLA-4, and promoting an immune-activating microenvironment, immunotherapy can induce long-term changes in the tumor ecosystem, reducing its ability to adapt and evolve in response to treatment. Additionally, combining immunotherapy with agents targeting the extracellular matrix or inhibiting angiogenesis can further destabilize the tumor microenvironment. For example, inhibiting angiogenesis not only deprives the tumor of nutrients but also alters the structural components that cancer cells rely on for growth and metastasis [70]. This combination of approaches could enhance the overall effectiveness of cancer treatments and reduce the likelihood of resistance. Moreover, recent advances in spatial transcriptomics and multiplexed imaging have provided new tools for studying how immunotherapies reshape the tumor microenvironment at the molecular level. These technologies enable researchers to identify specific cell–cell interactions and molecular signals within the tumor that could be targeted to disrupt its adaptive capacity. By integrating these insights, immunotherapy can be fine-tuned not only to target cancer cells but also to weaken the tumor’s support systems, making it more vulnerable to additional treatments.

### Predicting GPC evolution

Intra-tumoral heterogeneity is generally viewed as the consequence of random mutation and selection. However, specific patterns can be predicted. For instance, Carmona-Fontaine et al [71] showed that the altered metabolism of cancer cells can create predictable gradients of extracellular metabolites (e.g. oxygen, lactate) that organize the phenotypic composition of a tumor (i.e. tumor morphogens). Similarly, the vascular network of a tumor can affect the distribution of oxygen and metabolites that serve as predictable, environmental selection forces governing the distribution of cancer cells with various phenotypes [72]. Furthermore, the compositional and configurational heterogeneity of components in the tissue microhabitat can influence resource availability and functional connectivity and also play a crucial role in facilitating metastasis [73].

A tumor’s GPC is expected to change as the micro-environment changes. Similar to societies and ecosystems, only tumors that can change their GPCs but still maintain their oncogenic status will persist or recover in response to therapies. This system-level ecology-based rather than cell-based perspective should help predict tumor response to therapies. In the cell-based perspective, tumor regression is the result of targeted cells being affected, and recurrence is expected because of the surviving resistant cells. In the ecology-based perspective, it is the disruption of the functional networks that causes tumor regression. However, because the high mutational and plastic potential, some tumors can achieve new GPCs with new functional networks and new strategies to exploit and adapt to the new environment. Thus, an understanding of the evo-eco dynamics during relapse could help anticipate these reconfigurations and interfere with the re-establishment of an oncogenic GPC. Also, as surviving cells have to be plastic enough to diversify the population and generate new functional and cooperative strategies, targeting the molecular basis of tumor cell plasticity [74] and cooperation should avoid cancer relapse. Drugs like Vemurafenib (a BRAF inhibitor) can initially reduce plasticity in specific types of tumors, but combining BRAF inhibitors with MEK inhibitors, such as Dabrafenib with Trametinib or Vemurafenib with Cobimetinib, can help prevent the re-emergence of resistant clones by simultaneously targeting multiple survival pathways [75].

Predictive modeling of tumor response to sequential therapies could improve the design and timing of treatment regimens, allowing for more precise interventions (**see**  Box 5). Using biomarker-driven therapies, such as Trastuzumab for HER2-positive cancers or Olaparib for BRCA-mutant tumors, allows precision targeting of dominant cellular subsets whose activity may shape oncogenic GPCs. While these therapies act primarily at the cellular level, their systemic effects may also contribute to GPC disruption by weakening key functional interactions or cooperative dynamics within the tumor. These therapies can be further optimized through real-time monitoring of biomarkers and tumor evolution, allowing adjustments to therapy based on the emergence of new resistance mutations or alterations in GPCs. This adjustment in treatment sequencing could be crucial in outsmarting the tumor’s capacity for rapid GPC change, thus maintaining long-term control over its progression. With the implementation of sequential therapies (as discussed in Box **4**) combined with real-time monitoring of GPCs, a wealth of data will be generated. Over time, this data will enhance the dynamic adjustment of treatments and improve the ability to predict and anticipate GPC dynamics. Recent advances in artificial intelligence (AI) and machine learning algorithms have the potential to revolutionize real-time decision-making in cancer treatment [76]. These technologies can analyze vast amounts of data from genomic sequencing, imaging, and biomarker monitoring to predict tumor evolution and the emergence of resistant phenotypes. By continuously learning from patient-specific data, AI-driven platforms could recommend precise adjustments in therapeutic strategies, improving outcomes by anticipating GPC shifts and optimizing treatment timing and combinations. Integrating these tools into clinical practice could significantly enhance the precision of adaptive therapies, enabling a more dynamic, tailored approach to managing tumor progression and resistance. Specifically, information obtained from sequencing and imaging during the initial therapy phases can feed into advanced predictive models, facilitating the anticipation of GPC evolution and the adaptation of treatments accordingly. As previously proposed to fight cancer resistance due to cell-to-cell heterogeneity through systems biology approaches [77], we propose an iterative therapeutic strategy based on the repetitive characterization of subclonal phenotypic diversity and cell–cell interaction networks, followed by the reconstitution of the tumor GPCs and targeting of the most relevant nodes in the current GPCs. Advances in sequencing technologies will allow for the early identification of resistant subpopulations within the tumor, providing essential baseline data for ongoing monitoring. Similarly, magnetic resonance imaging and other cutting-edge technologies can track changes in the tumor microenvironment, shedding light on how GPCs change over time.

Box 5. Connecting model-based predictions with clinical applications to monitor and influence tumor progression in real-time.

GPCs represent clusters of functionally interacting cell populations that collectively optimize their response to environmental pressures, enhancing tumor resilience. Modeling these dynamics offers powerful insights into controlling tumor evolution through response to therapy.

**Modeling functional interactions within GPCs:** GPCs can be represented as networks of interconnected cells, with each node denoting a subpopulation contributing to a shared function (e.g. angiogenesis, immune evasion). The connections between nodes simulate cooperative behaviors, such as shared production of growth factors or immune-suppressive signals. To this extent, developing network models can assess how these interactions influence tumor robustness, the likelihood of resistance emergence and the weak points of these networks that could be targeted by therapy.

**Simulating GPC evolution under therapeutic pressure:** By incorporating evolutionary dynamics into population models, we can simulate how GPCs adapt to treatments. Therefore, analyzing the resulting population dynamics can be pivotal to predict which GPC configurations are likely to persist under different therapeutic pressures (like chemotherapy or immunotherapy). This approach allows for sequencing or adjusting treatments to ‘drive’ tumors into less aggressive states.

**Applications in adaptive therapy control strategies:** Connecting these models to clinical data may enable real-time monitoring of GPCs via liquid biopsies and spatial transcriptomics. While spatial transcriptomics provides a powerful window into the architecture and functional organization of solid tumors, it currently relies on invasive sampling and cannot yet be performed longitudinally in a clinically feasible manner. Therefore, its main utility lies in the foundational characterization of GPCs and their association with specific tumor functions or therapeutic responses. In contrast, liquid biopsies, especially those analyzing circulating tumor cells (CTCs), cell-free DNA (cfDNA), or extracellular vesicles, offer a non-invasive and repeatable approach better suited for real-time monitoring. Although they provide less spatial resolution, liquid biopsies can capture dynamic changes in the composition of circulating GPC-like structures (such as CTC clusters), potentially serving as a proxy for intratumoral evolution and therapeutic impact. Together, these technologies are complementary: spatial transcriptomics informs the construction of GPC models, while liquid biopsies offer a means to track their evolution and response to treatment in clinical settings. This surveillance informs adaptive strategies, such as ‘evolutionary double binds,’ to trap GPCs in less viable configurations. Combining such predictive modeling with cutting-edge machine learning algorithms allows to identify optimized protocols of iterative treatment adjustments to keep GPCs in a controlled, less aggressive state, potentially reducing the risk of relapse.

Modeling GPCs within the selection for function framework provides a novel approach for understanding and manipulating tumor evolution. By simulating functional adaptations and applying dynamic, targeted therapies, it could become feasible to slow or halt tumor progression through real-time, data-driven interventions.

Recent advances in molecular and spatial profiling technologies, particularly spatial transcriptomics, multiplexed imaging, and single-cell multi-omics, are now revolutionizing our capacity to move from a qualitative to a quantitative understanding of GPCs. Historically, the tumor microenvironment has been analyzed mainly through a descriptive or categorical lens, identifying key players or general patterns of cell interactions. In contrast, the concept of GPC requires a more integrative and quantitative approach, aiming to define not only the presence of specific cell types or molecular markers but also their spatial organization, interaction networks, and dynamic behaviors over time. Spatial omics technologies now allow for the precise mapping of cell–cell interactions, gradients of metabolites or signaling molecules, and the architecture of functional niches within tumors. These technologies provide essential data for constructing mathematical and computational models of GPC evolution, enabling simulation of tumor dynamics and the prediction of treatment responses under different therapeutic regimens. Importantly, while these approaches are still developing and may not be applicable in real-time in most clinical settings, they provide a powerful framework for refining our definition of GPCs and validating GPC-targeted therapeutic strategies in experimental and translational oncology.

By integrating these data into computational models and machine learning algorithms, predictions about the tumor’s GPC evolutionary trajectory can be refined, allowing clinicians to anticipate shifts in GPC dynamics and adjust treatments accordingly. Leveraging real-time data, these models enable more precise fine-tuning of therapeutic sequences, disrupting the tumor’s ability to optimize its GPC for survival. This proactive approach, driven by continuous data input, is vital for controlling tumor progression and overcoming its rapid adaptability.

### Practical challenges and guidelines for implementing GPC-based therapeutic strategies

While our framework offers new perspectives on tumor evolution and therapy, its practical implementation faces several challenges that require consideration. Capturing, monitoring, and predicting GPC dynamics in patients is inherently complex, due to limitations in current technologies, costs, and the feasibility of longitudinal multi-modal data collection. However, several emerging strategies could progressively enable this approach. First, defining relevant GPCs will require integrating multiple types of data, including spatial transcriptomics, single-cell analyses, and liquid biopsies to capture both the phenotypic heterogeneity and functional interactions of tumor cells over time. Importantly, not all modalities need to be captured simultaneously or continuously; a stepwise approach combining key biomarkers (e.g. signatures of cooperation, angiogenesis, immune evasion) with targeted functional assays may provide actionable proxies of GPC dynamics. Second, monitoring GPC evolution could rely on the iterative use of spatial and temporal sampling strategies (e.g. pre-treatment, during treatment, post-treatment) coupled with computational modeling and machine learning to infer likely trajectories and vulnerabilities. Third, predicting and validating GPC-based therapies, including sequential strategies, will require a shift towards adaptive trial designs. These trials would test the impact of disrupting functional networks or increasing internal conflicts within tumors, using combinations or sequences of existing drugs targeting cooperation, plasticity, or microenvironmental interactions. Finally, an important challenge will be to develop robust clinical decision-support tools that integrate heterogeneous data and help oncologists select the most appropriate treatment sequences based on predicted GPC vulnerabilities. While ambitious, this roadmap echoes advances already seen in precision oncology, immunotherapy, and eco-evolutionary treatment design.

Box 6. Summary and key avenues for future research.

***How do therapeutic approaches that take into account a tumor’s GPC differ from traditional cancer treatment strategies?***

Traditional cancer treatments (i.e. chemo/radiotherapy with or without prior resection) often aim to eliminate as many tumor cells as possible, focusing on targeting the fitness of individual cells. However, this approach may fail to address the complex functional networks within the tumor and may lead to treatment resistance. In contrast, GPC-based therapies aim to disrupt the tumor-level networks that enable tumor growth and persistence, such as disrupting angiogenesis, suppressing immune evasion, or altering the tumor microenvironment by acting simultaneously or successively on different components of the networks (e.g. key nodes, interactions among nodes and/or with the microenvironment).

***How can targeting networks within a tumor’s GPC lead to more effective therapeutic strategies?***

Targeting system-level networks involves identifying and disrupting specific pathways affecting cellular interactions that are essential for tumor growth and survival. For example, blocking signals that allow cancer cells to communicate and cooperate with each other and/or with stromal cells, or acclimate to environmental changes can inhibit tumor progression. This approach aims to destabilize the tumor by disrupting its organization and ability to adapt, making it more vulnerable to treatments.

***What role does the tumor microenvironment play in establishing an oncogenic GPC, and how can it be exploited for therapeutic purposes?***

The tumor microenvironment, which includes immune cells, fibroblasts, blood vessels, and the extracellular matrix, plays a crucial role in forming and maintaining an oncogenic GPC. Interactions between tumor cells and the microenvironment can either support or hinder tumor progression. Therefore, modifying the tumor microenvironment, such as altering the pH, inhibiting angiogenesis or enhancing the anti-tumor immune response, can potentially disrupt the oncogenic GPC and inhibit tumor growth.

***Why are sequential therapies promising for preventing GPC optimization and managing tumor progression?***

Tumors can rapidly evolve in response to treatments, leading to therapeutic resistance. Sequential therapies involve using different treatments or combinations of treatments in a specific and timed order to prevent tumor adaptation. By alternating treatments that target different tumor functions or components of the GPC, this approach aims to prevent the tumor from reaching a stable, resistant state, thus improving the long-term effectiveness of treatments.

***How does understanding ‘selection for function’ and GPC shape the future of cancer research and treatment?***

The concept of ‘selection for function’ and the recognition of the importance of GPC in tumor progression represent a paradigm shift in our understanding of cancer. It highlights the need for therapeutic approaches that go beyond targeting the fitness of individual cancer cells and instead focus on disrupting the tumor’s complex ecosystem and the evolution of its GPC. This perspective is expected to lead to the development of more effective and personalized treatment strategies aimed at controlling tumor progression and improving patient outcomes.

## CONCLUSION

Advances in drug development in the past few decades have introduced a wide range of new cancer treatment strategies and agents. While overall survival has increased, most common metastatic cancers remain fatal because of the remarkable ability of cancer cells to evolve resistance to all currently available treatments. Thus, it is not an exaggeration to view evolution as the ultimate cause of death in many, perhaps most, cancer patients. Consequently, understanding tumor progression through the lens of ‘selection for function’ [7] allows new treatment perspectives. Rather than attempting to destroy all tumor cells with a single (‘magic bullet’) treatment, the ‘selection for function’ paradigm seeks to manipulate the evolutionary dynamics of the tumor, through its GPC, to steer its evolutionary trajectory toward extinction or, at least, a less aggressive state. By combining therapies that attack multiple functional networks within the tumor, such as angiogenesis, immune evasion, and matrix remodeling, we could significantly reduce the chances of the tumor adapting and becoming resistant to treatment. While suppression of many of these interactions is already considered within conventional therapeutic development pipeline, consideration of GPC provides a distinct perspective, enabling consideration of therapeutic interventions that do not necessarily lead to therapeutic enhancement at short-term scales but enhance long-term therapeutic efficiencies by reducing the likelihood of resistance and enhancing the odds of cancer eradication. Rather than contradicting prior models of tumor evolution, our framework enriches them by highlighting the role of system-level ‘selection for function’ (i.e. for increased resilience and ability to progress). Our framework may also offer insight into a longstanding paradox: why cancer is relatively rare despite the enormous number of cell divisions occurring in multicellular organisms. While mutations and clonal expansions are frequent, only those nascent tumors that manage to stabilize functionally coherent group-level traits, i.e. oncogenic GPCs, are likely to persist and progress. From this perspective, the evolution of anti-cancer defenses may have targeted not only individual mutated cells, but also mechanisms that disrupt or prevent the formation of such group-level functional configurations. This adds a new dimension to our understanding of how multicellular organisms suppress cancer: by preventing the emergence or persistence of functional tumor-level architectures, not just by eliminating aberrant individual cells.

Our evolutionary approach does not dismiss the importance of mutations or the TME but adds a missing dimension to understanding why some tumors remain resilient and how therapies can be adapted to destabilize them at a more systemic level. This perspective has direct implications for therapy: rather than solely targeting individual oncogenic mutations or disrupting isolated tumor-microenvironment interactions, therapeutic strategies could focus on dismantling the functional networks that allow tumors to persist and adapt. By shifting the therapeutic target from individual cells to GPC dynamics, this approach could help overcome resistance and improve treatment durability. Ultimately, embracing this comprehensive approach to cancer treatment may lead to breakthroughs not only in therapeutic efficacy but also in reducing relapse rates. By intervening at the system network level, this strategy has the potential to deliver more durable outcomes and shift the treatment paradigm from temporary remission to long-term control of cancer progression. By adopting a more nuanced view of tumor behavior and leveraging advanced technologies, we could significantly enhance our ability to manage and ultimately overcome cancer. Embracing this comprehensive approach may lead to breakthroughs in both treatment and understanding, offering new hope for improved patient outcomes and survival (**see**  Box 6).

## GLOSSARY

- **Allee Group Dynamics**: A phenomenon in ecology where the fitness of individuals within a population is enhanced at higher densities. In the context of cancer, this means that certain tumor cells can benefit from being part of a larger group, as their collective presence may help them evade host immune responses or diminish the effectiveness of treatments. For example, increased cell density can lead to the production of signaling molecules that promote growth and survival, or it may create a microenvironment that provides protective factors against therapeutic agents. Understanding Allee group dynamics in tumors thus highlights the need for innovative therapeutic strategies that consider not only the individual characteristics of cancer cells but also their interactions and collective behaviors within the tumor microenvironment.
- **Cluster of Circulating Tumor Cells (CTC Cluster)**: An aggregation of circulating tumor cells in the bloodstream. Clusters of CTCs include homotypic clusters made of cancer cells only, as well as heterotypic clusters that incorporate stromal or immune cells along with cancer cells. Unlike single CTCs, these clusters exhibit emergent properties that enhance their metastatic potential, particularly by facilitating immune evasion and survival in circulation.
- **Evo-eco Perspective**: An approach combining principles of ecology and evolution to understand tumor dynamics. This perspective allows for analysis of how interactions within tumors and with their microenvironment influence tumor evolution and resilience.
- **Evolutionary Double Bind**: A therapeutic strategy aimed at trapping cancer cells in a situation where adaptations that make them resistant to one treatment render them vulnerable to another. This approach relies on treatment sequences to impose evolutionary constraints, limiting the tumor’s adaptive options.
- **Tumor-level Network:** An integrated set of cellular behaviors and interactions within a tumor that collectively support a particular emergent property, such as invasion, or immune evasion. Such networks reflect not only the presence of certain cell types, but also the organization, spatial distribution, and interactions among these cells. Disrupting key networks can compromise tumor stability, even if individual cells remain viable.
- **Group-Level Trait:** A trait or behavior that emerges from the collective action of multiple cells and contributes to the resilience and progression of the tumor as a system. Examples include shared metabolic networks, cooperative immune evasion, or matrix remodeling. Such traits are not meaningful at the level of isolated cells but are essential for the tumor’s progression.
- **Group Phenotypic Composition (GPC)**: GPC refers to the emergent, spatially and functionally organized configuration of diverse cellular subpopulations within a tumor, whether cancerous, stromal, or immune, that collectively contribute to tumor-level emergent properties such as angiogenesis, immune evasion, or extracellular matrix remodeling. Unlike earlier definitions emphasizing shared (epi)genotypes, we define GPCs primarily by their contribution to tumor persistence and progression, irrespective of clonal lineage. GPCs are shaped by local microenvironmental conditions and can reconfigure dynamically in response to selective pressures such as therapy. A GPC is considered *oncogenic* if it promotes tumor persistence and ability to adapt (i.e. to change its GPC) in a given context.
- **Liquid Biopsy**: A non-invasive sampling method that detects and analyzes circulating tumor cells, circulating tumor DNA, extracellular vesicles or other circulating biomarkers in blood or other body fluids. It is particularly useful for tracking GPC evolution and treatment responses in real-time.
- **Selection for Function**: A process by which system configurations that enhance the static and dynamic persistence of a system as well as its ability to generate novelty are favored, (see Wong et al. [12]). Unlike classical Darwinian selection, this form of selection can favor systems with configurations that can emerge *de novo*, rather than relying on heritability. In tumors, it helps explain the emergence and maintenance of group-level properties (e.g. angiogenesis, immune evasion, stromal remodeling).
- **Spatial Transcriptomics**: A technology that maps gene expression of cells in their spatial context within a tissue. Applied to tumors, this technology helps visualize cellular interactions in GPCs and identify specific therapeutic targets.
- **System-Level Therapy:** A therapeutic approach targeting the emergent properties and organizational features of tumors, such as cooperation, division of labor, or ecological dependencies, rather than individual genetic mutations or cell-autonomous traits. These strategies aim to destabilize oncogenic GPCs, impair functional integration, and push the tumor past a systemic tipping point.
- **Tipping Point:** A critical threshold in tumor dynamics at which a small perturbation (e.g. therapeutic intervention) can lead to a major shift in the system’s behavior, such as collapse of the tumor’s organization or reversion to a less aggressive state. GPCs may display tipping points when their internal coordination or external support is destabilized beyond repair.
